# PD-1 inhibitor versus bevacizumab in combination with platinum-based chemotherapy for first-line treatment of advanced lung adenocarcinoma: A retrospective-real world study

**DOI:** 10.3389/fonc.2022.909721

**Published:** 2022-11-09

**Authors:** Zhe Huang, Chunhua Zhou, Yi Xiong, Feng Yang, Fanxu Zeng, Wenjuan Jiang, Yongchang Zhang, Haiyan Yang, Li Liu, Liang Zeng, Nong Yang, Zhan Wang

**Affiliations:** ^1^ Department of Medical Oncology, Lung Cancer and Gastrointestinal Unit, Hunan Cancer Hospital/The Affiliated Cancer Hospital of Xiangya School of Medicine, Central South University, Changsha, China; ^2^ Graduate Collaborative Training Base of Hunan Cancer Hospital, Hengyang Medical School, University of South China, Hengyang, China; ^3^ Center of New Drug Clinical Trial, Hunan Cancer Hospital and The Affiliated Cancer Hospital of Xiangya School of Medicine, Central South University, Changsha, China

**Keywords:** bevacizumab, chemotherapy, immune checkpoint inhibitors, NSCLC, over-all survival

## Abstract

**Background:**

Chemotherapy combined with immunotherapy or anti-vascular therapy is both recommended by guidelines for first-line treatment of lung adenocarcinoma. However, no head-to-head clinical trial has ever compared which strategy is the optimal choice. This real-world retrospective study was done to compare the efficacy and treatment-related adverse events of immunotherapy and bevacizumab in combination with chemotherapy.

**Patients and methods:**

From January 2018 to March 2021, we retrospectively collected 276 patients with advanced lung adenocarcinoma managed with chemotherapy combined with bevacizumab or PD-1 inhibitors at our center. Among them, 139 patients were treated with chemotherapy combined with bevacizumab, while 137 patients were treated with chemotherapy combined with PD-1 inhibitors. After receiving four cycles of combination therapy, all patients received maintenance therapy until disease progression. Progression‐free survival (PFS), overall response rate (ORR), overall survival (OS), disease control rate (DCR), and adverse events (AE) were analyzed between the two groups.

**Results:**

Compared to patients who received anti-vascular therapy, patients who underwent immunotherapy achieved better PFS (7.3 months vs. 10 months, p = 0.002) while ORR (40.9% vs. 51.1%, p = 0.093), as well as OS (18 months vs. 24 months, p = 0.060), had no statistical difference between the two groups. In the PD-L1-negative population, there was no statistical difference in PFS and OS between the two groups. (8.0 months VS. 6.0 months, p = 0.738; and 19 months vs. 13 months, p = 0.274). In the PD-L1-positive population, there was a significant benefit in PFS in the population receiving immunotherapy (7.0 months vs. 10.0 months, p = 0.009). Proteinuria and hypertension occurred more frequently in the bevacizumab-treated group (p = 0.001 and p = 0.002), whereas immune-related pneumonia and hypothyroidism occurred more frequently in the immunotherapy-treated group (p = 0.007 and p = 0.030).

**Conclusions:**

The addition of a PD-1 inhibitor was superior to bevacizumab in terms of PFS among patients with advanced lung adenocarcinoma. PD-L1-positive patients appeared to exhibit better PFS, OS, and ORR. Toxic reactions were manageable in both groups.

## Introduction

Lung cancer is the highest incidence of cancers in men and the leading cause of cancer-related deaths worldwide (1, 2). The treatment landscape for advanced, unresectable, and/or metastatic non-small cell lung cancer (NSCLC) is evolving. The standard of care for patients with driver mutation-negative metastatic lung adenocarcinoma included the combination of platinum-doublet chemotherapy with bevacizumab or immune checkpoint inhibitors (ICIs) (3). Bevacizumab exerts an effective antitumor effect by targeting and inhibiting human vascular endothelial growth factor, promoting the normalization of tumor vessels, and reducing the formation of new blood vessels. The combination of bevacizumab plus chemotherapy (B + C) is a formally approved intervention in unselected patients except those with treatment-related contraindications (4). The anti-angiogenic therapy has greatly improved and to a certain extent, prolonged the survival time of patients and improved their quality of life (5–8).

The monotherapy of ICIs (anti-programmed death 1 PD-1) has been shown to provide an overall survival benefit for selected NSCLC patients who have programmed death ligand 1 (PD-L1) expression on at least 50% of tumor cells (9, 10). The combination of chemotherapy and ICIs (I + C) improves survival regardless of PD-L1 status and results in a higher ORR than monotherapy (11, 12). Although integrating immunotherapy into a treatment plan for NSCLC improved survival and quality of life for some patients, predictive biomarkers for ICIs are still under investigation. What is certain is that some oncogenetic alterations in tumors, such as EGFR or ALK, show poor response to ICI treatment and are associated with an increased occurrence of toxic effects (13, 14). Therefore, the initial treatment for NSCLC patients with EGFR or ALK genetic alterations should be target therapy. The ICI agents (PD-1/PD-L1 inhibitors) are recommended by the NCCN guidelines for first-line treatment of driver mutation-negative advanced NSCLC (15–17).

However, it is inconclusive whether chemotherapy combined with bevacizumab (B + C) or chemotherapy combined with immunotherapy (I + C) is optimal for patients with negative driver mutations in lung adenocarcinoma because of a lack of head-to-head trials. In the IMpower150 study, bevacizumab in combination with chemotherapy showed significant efficacy, and the overall survival benefit was not significantly inferior to atezolizumab plus chemotherapy but was significantly inferior to the addition of atezolizumab to bevacizumab and chemotherapy (ABC) (18). Moreover, in a number of network meta-analyses, B + C can be an optimal strategy as an initial first-line treatment for PD-1 positive advanced non-squamous NSCLC, while there is no detailed disadvantage compared with pembrolizumab treatment (19).

This retrospective cohort study aims to explore the efficacy and safety of chemotherapy combined with either bevacizumab or immunotherapy for first-line treatment of lung adenocarcinoma in a real-world setting to fill the gap in this regard.

## Patients and methods

### Patients

We retrospectively analyzed 2,522 treatment-naïve patients who were diagnosed with advanced lung adenocarcinoma from January 2018 to January 2022 at the Hunan Cancer Hospital. Patients with EGFR mutations, ALK fusions, or ROS-1 fusions were excluded. A total of 276 patients who were eligible for inclusion and received chemotherapy combined with bevacizumab or ICI were analyzed (**Figure S1**). All patients were ≥18 years old and histologically diagnosed with lung adenocarcinoma with stages III–IV. All patients had an Eastern Cooperative Oncology Group (ECOG) performance status (PS) of 0–2. The characteristics of the patients, including sex, age, smoking history, brain metastasis, liver metastasis, bone metastasis, and gene mutation status, are summarized in **Table 1**. For the classification of concomitant gene mutations, we referred to the results of the BENEFIT study by Jie et al. (20). All procedures in our study were performed in accordance with the ethical standards of the institutional and national research committees and 2013 revised Declaration of Helsinki. It was approved by the Ethics Committee of Hunan Cancer Hospital (approval number: 2017YYQ-SSB-026).

**Table 1:** Characteristics of patients in this study.

	Chemo + BEV (n = 137)	Chemo + ICIs (n = 139)	p-value
**Age [median (range), year)]**	60 (37–74)	59 (33-79)	0.109
**Gender**			0.657
Male	107 (78.1)	112 (80.6)	
Female	30 (21.9)	27 (19.4)	
**Smoking**			0.692
Non-smoker	42 (30.7)	39 (28.1)	
Former smoker	95 (69.3)	100 (71.9)	
**ECOG PS**			1.000
Low (0–1)	133 (97.1)	134 (96.4)	
High (2–3)	4 (2.9)	5 (3.6)	
**Stage**			0.521
IIIB–C	13 (9.5)	10 (7.2)	
IV	124 (90.5)	129 (92.8)	
**Brain metastasis at baseline**			0.514
With	24 (17.5)	20 (14.4)	
Without	113 (82.5)	119 (85.6)	
**Liver metastasis at baseline**			0.174
With	24 (17.5)	16 (11.5)	
Without	113 (82.5)	123 (88.5)	
**Bone metastasis at baseline**			0.549
With	61 (44.5)	67 (48.2)	
Without	76 (55.5)	72 (51.8)	
**Gene mutation**			0.282
None	66 (48.2)	54 (38.8)	
Multi-drive mutation	57 (41.6)	66 (47.5)	
Tumor-suppress mutation	14 (10.2)	19 (13.7)	

### Treatment

For this retrospective study, all patients who received induction treatment were administered on day 1 of each 21-day period: the regimen of cisplatin (75 mg/m^2^)/carboplatin (area under the curve, AUC 6), pemetrexed (500 mg/m^2^) and (7.5 or 15 mg/kg) bevacizumab (B + C) or cisplatin/carboplatin plus pemetrexed and PD-1 inhibitors (I + C). The prescription of PD-1 inhibitors in this study included pembrolizumab (n = 65) and sintilimab (n = 74), with a fixed dose of 200 mg. Induction chemotherapy was repeated every 3 weeks for a maximum of four cycles. After completion of at least three cycles of induction chemotherapy, patients received maintenance chemotherapy on day 1 of the 21-day cycle comprising pemetrexed with either bevacizumab or ICIs until the occurrence of unmanageable toxic effects or disease progression.

### Assessment

Chemotherapy response was evaluated after every two treatment cycles by computed tomography (CT). They were evaluated as complete response (CR), partial response (PR), stable disease (SD), and progression disease (PD) according to the Response Evaluation Criteria in Solid Tumor Criteria 1.1.9. The objective remission rate (ORR) was defined as the sum of CR and PR. The disease control rate (DCR) was defined as the sum of CR, PR, and SD. Toxicities were assessed according to the National Cancer Institute Common Terminology Criteria for Adverse Events version 5.0. The primary endpoints were PFS and ORR. Secondary endpoints were overall survival (OS), DCR, and adverse effects (AEs).

### Statistics analysis

Descriptive summaries were created for demographic and clinical variables. The chi-squared test was used to compare subset variables and toxicities. All *p*-values were two-tailed. Kaplan–Meier curves were generated for progression-free survival and overall survival. Log-rank tests were used to compare the survival between groups. All statistical analyses were performed using the SPSS 26.0 software for Windows (SPSS Corp., Armonk, NY, USA); *p <*0.05 was considered to indicate a statistically significant difference.

## Results

### Patient characteristics

A retrospective analysis was performed on 276 lung adenocarcinoma patients who had received first-line treatment. A total of 137 patients received chemotherapy combined with bevacizumab, and 139 patients received chemotherapy with PD-1 inhibitors. All the patients were without driver mutations. The characteristics of the patients, including sex, age, smoking history, brain metastasis, liver metastasis, bone metastasis, and gene mutation status, are summarized in **Table 1**. There were no significant differences in the baseline characteristics. According to the TNM classification for NSCLC patients (AJCC 7th). All patients had locally advanced or advanced lung adenocarcinoma.

### Clinical efficacy

**Figure 1:**
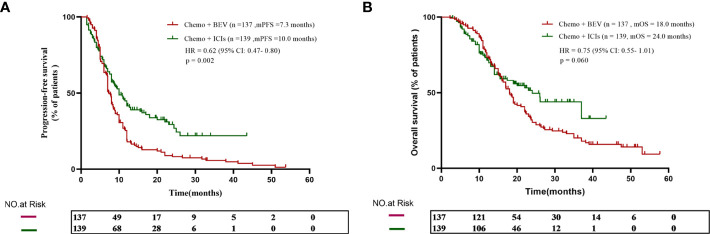
**(A)** The progression-free survival curve of patients who received chemotherapy plus either bevacizumab or ICIs. **(B)** The overall survival curve of patients who received chemotherapy plus either bevacizumab or ICIs.

Patients who received B + C achieved an mPFS of 7.3 months, while patients who received I + C achieved an mPFS of 10.0 months. The I + C group’s progression-free survival was longer (HR = 0.62, 95% CI: 0.47–0.80, *p* = 0.002, **Figure 1A**). The mOS was 18.0 months in the B + C group and 24.0 months in the I + C group. There was a prolonged OS observed in patients in the I + C group, although the difference was not statistically significant (HR = 0.75, 95% CI: 0.55–1.01, *p* = 0.060, **Figure 1B**).

The treatment responses are listed in **Table 2**. There was no patient who achieved CR in the whole population. Of the 137 patients in the treatment of the B + C group, 56 (40.9%) achieved PR, 68 (49.6%) achieved SD, and six (9.3%) showed PD, resulting in an ORR of 40.9% and a DCR of 90.5%. Of the 139 patients in the treatment of the I + C group, 71 (51.1%) achieved PR, 52 (37.4%) achieved SD, and 16 (11.5%) showed PD, resulting in an ORR of 51.1% and a DCR of 88.5%. There is no significant difference in ORR (p = 0.093) and DCR (p = 0.695) between the two groups.

**Table 2:** Treatment response.

	Chemo + BEV (n = 137)	Chemo + ICIs (n = 139)	p-value
CR	0	0	
PR	56 (40.9)	71 (51.1)	
SD	68 (49.6)	52 (37.4)	
PD	13 (9.5)	16 (11.5)	
ORR	56 (40.9)	71 (51.1)	0.093
DCR	124 (90.5)	123 (88.5)	0.695

CR, complete response; PR, partial response; SD, stable disease; PD, progression disease; ICI, immune checkpoint inhibitor; BEV, bevacizumab.

**Figure 2:**
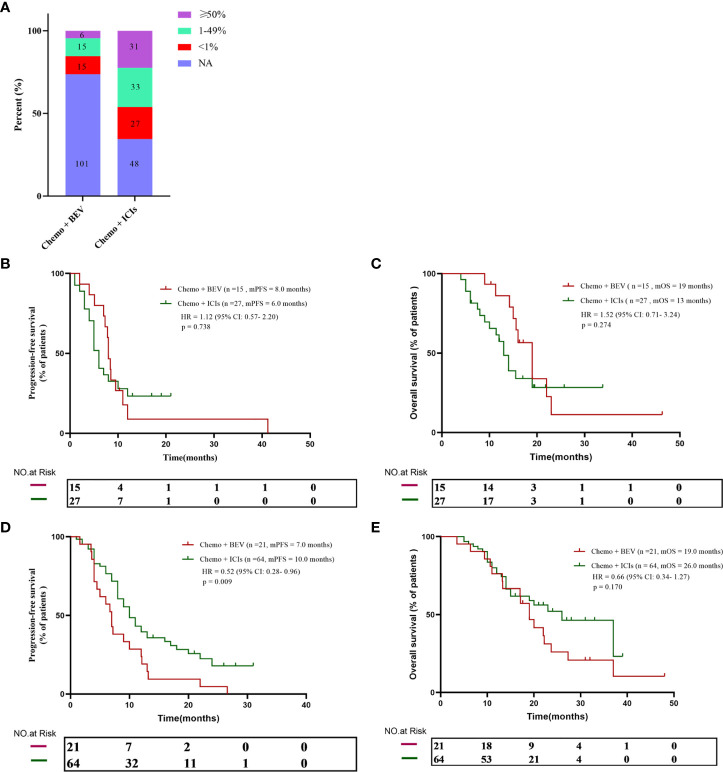
**(A)** PD-L1 expression in all patients. **(B, C)** The progression-free survival and overall survival curve of PD-L1 negative patients received chemotherapy plus either bevacizumab or ICIs. **(D, E)** The progression-free survival and overall survival curve of PD-L1 positive patients who received chemotherapy plus either bevacizumab or ICIs.

Considering that tumor PD-L1 expression is an important biomarker for immunotherapy, we further analyzed the relationship between PD-L1 expression and prognosis in the population. In the B + C group, only 36 patients were tested for PD-L1, including 15 PD-L1-negative patients, 15 low-expressing patients, and six high-expressing patients. In the I + C group, 91 patients’ PD-L1 expression status was available, including 27 negative patients, 33 patients with low expression, and 31 patients with high expression (**Figure 2A**). Next, we further divided the population by PD-L1 expression level to analyze the treatment effect in different populations. We found that there was no statistical difference between PFS and OS in the two groups in the PD-L1-negative population (8.0 months vs. 6.0 months, p = 0.738; and 19 months vs. 13 months, p = 0.274) (**Figures 2B, C**). However, in the PD-L1-positive population, the I + C group achieved a significantly better PFS (7.0 months vs. 10.0 months, p = 0.009) (**Figure 2D**). Although there was no statistical difference in OS between the two groups, there was still a sustained benefit for patients in the I + C group (19 months vs. 26 months, p = 0.170) (**Figure 2E**).

**Figure 3:**
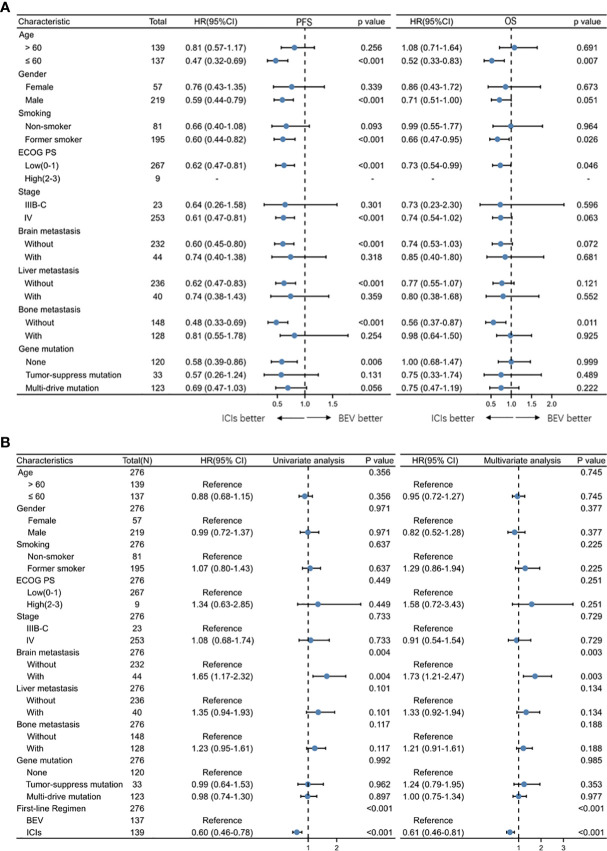
**(A)** Forest plots of hazard ratios for progression-free survival by subgroup for BEV + Chemotherapy and ICI + Chemotherapy group. **(B)** Univariate and multivariate Cox regression analysis of clinical characteristics of all patients.

An exploratory subgroup analysis of survival time was conducted, which was based on patients’ initial different characteristics. We found that in most patients, immunotherapy achieved better PFS. Consistent with previous results, OS was not statistically different between the two treatment modes for most patients. Immunotherapy has a better OS in patients younger than 60 years old, with a PS score of 0–1, smoking, as well as in patients without initial brain metastases (**Figure 3A**). In univariate and multivariate Cox regression analyses of PFS, the addition of ICI was a protective factor, whereas in patients with initial brain metastases it was a poor prognostic factor (**Figure 3B**).

### Toxicity

The most common grade I/II adverse events in the B + C group were leukopenia (n = 14, 10.2%) and liver injury (n = 14, 10.2%). In the I + C group, it was liver injury (transaminases increased) (n = 16, 11.5%). We found that these adverse events were mostly related to chemotherapy, resulting in no statistical difference between the two groups. Proteinuria occurred in 10 patients (7.3%) and hypertension in nine patients (6.6%) in the B + C group, which did not occur in the I + C group (p = 0.001 and p = 0.002) and was considered to be a bevacizumab-specific adverse event. In the immunotherapy group, immune pneumonitis occurred in eight patients (5.8%) and hypothyroidism in six patients (4.3%), which were not present in the bevacizumab treatment group, considering the unique adverse events of immunotherapy (P = 0.007 and p = 0.030). Similarly, there was no statistical difference in the incidence of grade III/IV adverse events between the two groups (**Table 3**, **Figure S2**).

**Table 3:** Adverse events.

	Grades 1–2		p value	Grades 3–4		p value
	Chemo + BEV (n = 137)	Chemo + ICIs (n = 139)		Chemo + BEV (n = 137)	Chemo + ICIs (n = 139)	
Leukopenia	14 (10.2)	1 (0.7)	<0.001	7 (5.1)	2 (1.4)	0.102
Pneumonia	0	8 (5.8)	0.007	0	0	–
Transaminases increased	14 (10.2)	16 (11.5)	0.847	0	1 (0.7)	1.000
Enteritis	0	0	–	0	1 (0.7)	1.000
Fatigue	4 (2.9)	1 (0.7)	0.212	0	0	–
Appetite Decreased	3 (2.2)	2 (1.4)	0.683	0	0	–
Rash	0	1 (0.7)	1.000	0	1 (0.7)	1.000
Hypothyroidism	0	6 (4.3)	0.030	0	0	–
Vomiting	2 (1.5)	0	0.245	6 (1.4)	2 (1.4)	0.171
Myositis	0	1 (0.7)	1.000	0	0	–
Bilirubin increased	0	3 (2.2)	0.247	0	0	–
Hepatitis	0	1 (0.7)	1.000	0	1 (0.7)	1.000
Anemia	8 (5.8)	2 (1.4)	0.059	2 (1.5)	2 (1.4)	1.000
Thrombocytopenia	2 (1.5)	1 (0.7)	0.621	1 (0.7)	2 (1.4)	1.000
Hypopituitarism	0	1 (0.7)	1.000	0	0	–
Myocarditis	0	0	–	0	1 (0.7)	1.000
Proteinuria	10 (7.3)	0	0.001	0	0	–
Hemoptysis	2 (1.5)	0	0.245	0	0	–
Hypertension	9 (6.6)	0	0.002	3 (2.2)	0	0.121
Epistaxis	1 (0.7)	0	0.496	0	0	–
Insomnia	2 (1.5)	0	0.245	0	0	–
Thrombosis	1 (0.7)	0	0.496	0	0	–
Constipation	4 (2.9)	0	0.059	0	0	–
Hematochezia	1 (0.7)	0	0.496	1 (0.7)	0	0.496

## Discussion

Immunotherapy has become an important therapy for advanced cell lung cancer, and a variety of immune checkpoint inhibitors have been approved for the first-line treatment of lung cancer (11, 15, 16, 18, 21). Especially in patients with driver gene-negative non-squamous NSCLC, multiple clinical trials have confirmed that immunotherapy not only improves the disease response rate but also prolongs survival compared with chemotherapy, largely improving the treatment outcome of advanced lung cancer. Meanwhile, bevacizumab combined with platinum-based doublet chemotherapy is the recommended regimen for first-line treatment of non-squamous non-small cell lung cancer and prolongs the survival time of patients compared with chemotherapy (7, 22–25). There is no study comparing the efficacy of the addition of PD-1/L1 inhibitors or bevacizumab to chemotherapy, and the question of whether PD-L1 negative patients should receive B + C remains controversial. There is rapidly evolving evidence showing the data of different combination strategies. In the IMpower150 study, the overall survival of chemotherapy combined with bevacizumab was not significantly inferior to atezolizumab combined with chemotherapy (19 months vs. 15 months, p = 0.07) (18). In the final overall survival analysis of IMpower150, in the PD-L1-negative subgroups, no difference in OS was observed with each combination subgroup (26). With meta-analyses, we have demonstrated that in non-squamous NSCLC with PD-L1 ≥50%, B + C was similar to pembrolizumab alone in terms of PFS. With PD-L1 <50%, the ICIs plus chemotherapy performed only marginally better than B + C (19). As far as we know, there are few real-world studies for comparison of the first-line PD-1 inhibitor versus bevacizumab in combination with chemotherapy directly.

The population of our study was patients with advanced or locally advanced lung adenocarcinoma, and the pathological types were consistent. We observed a significant PFS benefit in IC, somewhat different from the results of the previous IMpower150 study. This may be relevant to our population of patients selected for lung adenocarcinoma alone and without driver mutations. In addition, the PD-1 inhibitor we used for immunotherapy may be somewhat different from atezolizumab, while the chemotherapeutic drugs pemetrexed and paclitaxel may also be somewhat different. In this real-world study, time-to-event outcomes for each group were consistent with most published data on similar treatment strategies in clinical trials (12, 27, 28). Although there was no statistically significant difference in OS between the two groups, the benefit of the immunotherapy group was evident, and the conclusion was consistent with the study results of IMpower150 (26). More interestingly, we found an association between the expression level of PD-L1 and treatment modalities. The ORR for Bev with PEM/CARBP (40.9%) in our study was higher than that of the POINTBREAK study (34%). This may have reduced the magnitude of the benefit of ICI+ chemo (29). Compared with PFS, treatment beyond first-line progression had an impact on the analysis of OS. More interestingly, we found an association between the expression level of PD-L1 and treatment modalities. In the PD-L1-negative population, there was no significant difference in PFS and OS between the two groups. In PD-L1-positive patients, PFS was beneficial in patients receiving immunotherapy. This result was consistent with Impower 150 analysis data (26). However, due to the limited number of patients receiving PD-L1 testing in the B + C group (36/137), we did not observe the OS benefit in the I + C group. **Table S1** shows the results of comparing the use of chemo + ici *vs*. chemo + bev, which were cited from three meta-analyses and IMpower150.

In our study, there was no statistically significant difference in adverse events between groups. However, adverse events like those specific to bevacizumab, such as the occurrence of proteinuria, were not balanced between groups (*p* = 0.001). Similarly, such as rash, pneumonia, and enteritis, these phenomena were only observed in the ICI group. Fortunately, numerous treatment-related adverse events were controlled after certain management.

The limitations of our study include its retrospective nature and small sample size. A larger multi-center prospective study is needed to further confirm our findings. Moreover, among patients in the B + C group, the population for PD-L1 testing was too small, which affected the analysis results. In addition, OS was not reached in the immunotherapy arm due to the length of follow-up.

In conclusion, our study provides clinical evidence for the effectiveness of ICIs and bevacizumab in treating patients with advanced lung adenocarcinoma. In our study, ICI therapy resulted in a higher PFS, OS, and ORR. In PD-L1-negative patients, chemotherapy combined with bevacizumab was not inferior to immunotherapy, and in PD-L1-positive patients, immunotherapy was clearly superior. Toxicities were manageable in both groups.

## Data availability statement

The raw data supporting the conclusions of this article will be made available by the authors, without undue reservation.

## Ethics statement

The studies involving human participants were reviewed and approved by the Ethics Committee of Hunan Cancer Hospital (approval number: 2017YYQ-SSB-026). Written informed consent from the patients/participants or patients/participants’ legal guardian/next of kin was not required to participate in this study in accordance with the national legislation and the institutional requirements.

## Author contributions

(I) Conception and design: ZW and NY. (II) Administrative support: ZW, CZ, and NY. (III) Provision of study materials or patients: ZH, FY, and CZ. (IV) Collection and assembly of data: ZH, FY, YZ, YX, FZ, LL, WJ, and HY. (V) Data analysis and interpretation: ZW, ZH, and YZ. (VI) Manuscript writing and editing: ZW and ZH. All authors contributed to the article and approved the submitted version.

## Funding

This project were supported by the Hunan Provincial Natural Science Foundation of China (2021JJ40325, 2020JJ9044, 2020NSFC-B006, and 2020JJ9043), the Changsha Municipal Natural Science Foundation (kq2014208), and the Sailing Youth Fund of Hunan Cancer Hospital (2020QH002).

## Conflict of interest

The authors declare that the research was conducted in the absence of any commercial or financial relationships that could be construed as a potential conflict of interest.

## Publisher’s note

All claims expressed in this article are solely those of the authors and do not necessarily represent those of their affiliated organizations, or those of the publisher, the editors and the reviewers. Any product that may be evaluated in this article, or claim that may be made by its manufacturer, is not guaranteed or endorsed by the publisher.

## Supplementary material

Supplementary Figure 1 Flow diagram of the study design. BEV: bevacizumab. ICIs: immune checkpoint inhibitors

Supplementary Figure 2 Comparison of Adverse Events in B+C group and I+ C group
