# Supplementary material for: PD-1 inhibitor versus bevacizumab in combination with platinum-based chemotherapy for first-line treatment of advanced lung adenocarcinoma: A retrospective-real world study
Source: Front Oncol. 2022 Nov 9;12:909721. doi: 10.3389/fonc.2022.909721 (PMC9683483; doi:10.3389/fonc.2022.909721)
Supplement: Supplementary file 1 [file Table_1.doc]

| Table S1. Relevant systematic review and study for comparison of B+C and I+C, which are listed as following | | | | |  |
| --- | --- | --- | --- | --- | --- |
| Trail | Population | Subgroup | ORR | PFS | OS |
| Hui Yu, MA, 2021/10 | non-sq NSCLC | PD-L1 expression; Age; sex; PS | HR= 1.02, 95%CI 0.79 to 1.33; p=0.85 | HR= 0.78, 95% CI 0.60 to 1.00; p=0.059 | HR= 0.82, 95% CI: 0.71 to 0.94; p<0.01 |
| Andrew M Frederickson, NMA 2019/2 | Metastatic NSCLC | PD-L1 expression; Age; sex; PS | / | HR= 0.61, 95%CI: 0.36 to 1.01, P=0.065 | HR= 0.53, 95% CI: 0.35 to 0.79; P< 0.01 |
| Our study | Lung adenocarcinoma | PD-L1 expression; Age; sex; PS | HR= 0.80, 95%CI 0.61 to 1.04; p=0.093 | HR= 0.62, 95%CI:0.47-0.80, p= 0.002 | HR= 0.75, 95% CI: 0.55 to 1.01; P = 0.060 |
| MA, meta-analysis; NMA, network meta-analysis; ORR, objective response rate; PFS, Progression-free survival; OS, overall survival. | | | | | |
