# Supplementary figures and images for: PD-1 inhibitor versus bevacizumab in combination with platinum-based chemotherapy for first-line treatment of advanced lung adenocarcinoma: A retrospective-real world study
Source: Front Oncol. 2022 Nov 9;12:909721. doi: 10.3389/fonc.2022.909721 (PMC9683483; doi:10.3389/fonc.2022.909721)

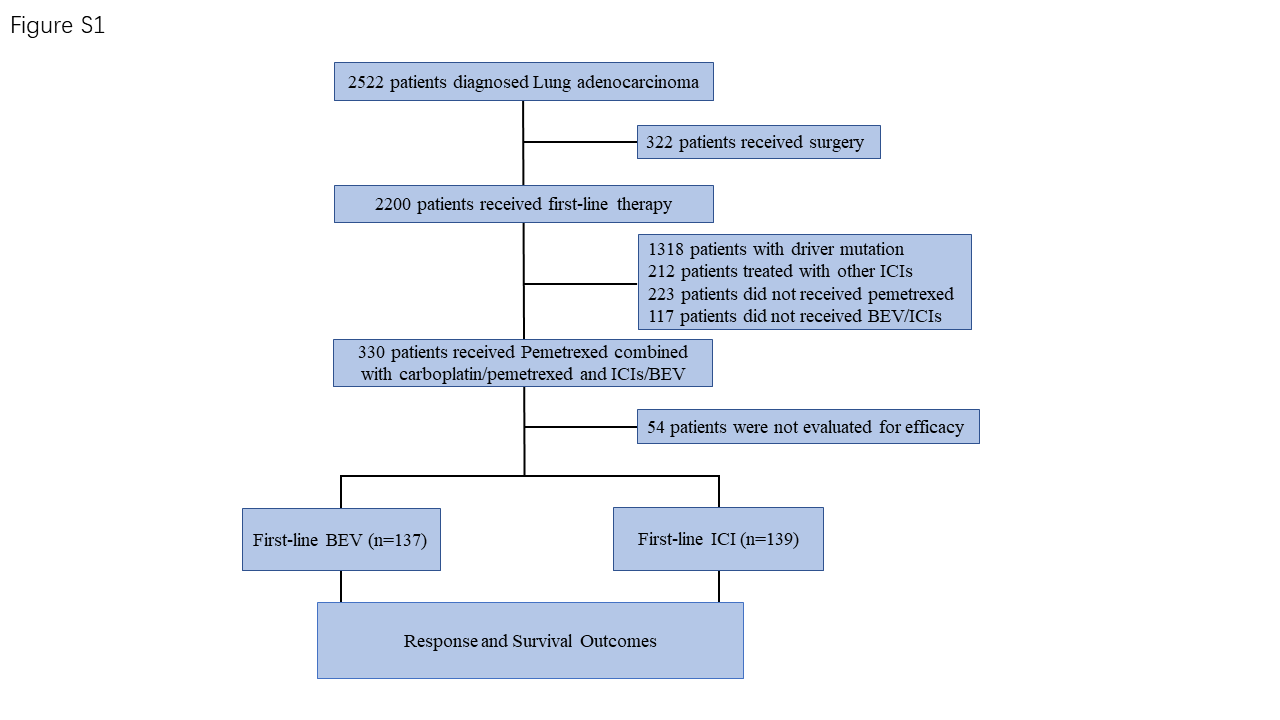

Supplement: Supplementary Figure 1 — Flow diagram of the study design. BEV: bevacizumab. ICIs: immune checkpoint inhibitors [file Image_1.tif]

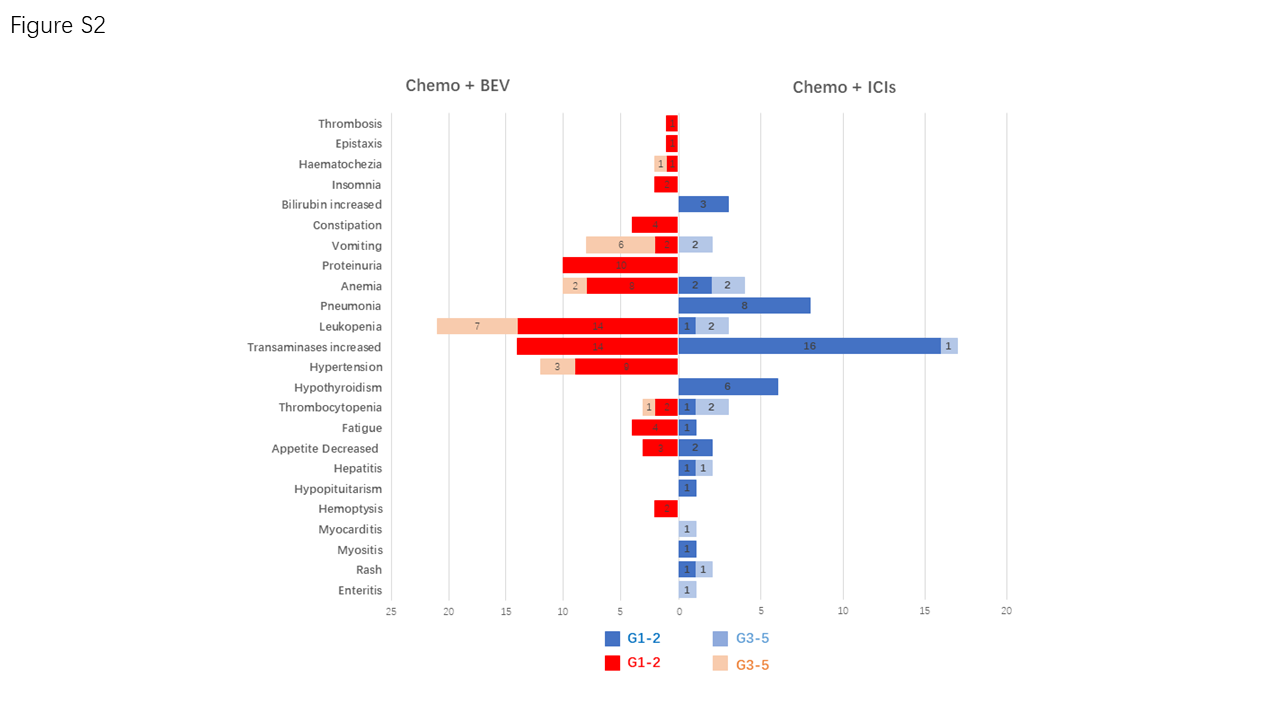

Supplement: Supplementary Figure 2 — Comparison of Adverse Events in B+C group and I+ C group [file Image_2.tif]
